# Effects of social network diversity in the disablement process: a comparison of causal inference methods and an outcome-wide approach to the Indonesian Family Life Surveys, 2007–2015

**DOI:** 10.1186/s12939-020-01238-9

**Published:** 2020-07-31

**Authors:** Julia Schröders, Fatwa Sari Tetra Dewi, Maria Nilsson, Mark Nichter, Miguel San Sebastian

**Affiliations:** 1 Department of Epidemiology and Global Health, Umeå University, Umeå, Sweden; 2 Department of Health Behaviour, Environment and Social Medicine, Universitas Gadjah Mada, Yogyakarta, Indonesia; 3 School of Anthropology, University of Arizona, Tucson, AZ USA

**Keywords:** Social network diversity, Aging populations, Disablement process, Non-communicable diseases, Cognitive function, Outcome-wide epidemiology, Propensity score matching, Instrumental variable, Causal inference, Lower-middle income countries

## Abstract

**Background:**

Social networks (SN) have been proven to be instrumental for healthy aging and function as important safety nets, particular for older adults in low and middle-income countries (LMICs). Despite the importance of interpreting health outcomes in terms of SN, in many LMICs – including Indonesia – epidemiological studies and policy responses on the health effects of SN for aging populations are still uncommon. Using outcome-wide multi-method approaches to longitudinal panel data, this study aims to outline more clearly the role of SN diversity in the aging process in Indonesia. We explore whether and to what degree there is an association of SN diversity with adult health outcomes and investigate potential gender differences, heterogeneous treatment effects, and effect gradients along disablement processes.

**Methods:**

Data came from the fourth and fifth waves of the Indonesian Family Life Survey fielded in 2007–08 and 2014–15. The analytic sample consisted of 3060 adults aged 50+ years. The primary exposure variable was the diversity of respondents’ SN at baseline. This was measured through a social network index (SNI), conjoining information about household size together with a range of social ties with whom respondents had active contact across six different types of role relationships. Guided by the disablement process model, a battery of 19 outcomes (8 pathologies, 5 impairments, 4 functional limitations, 2 disabilities) were included into analyses. Evidence for causal effects of SN diversity on health was evaluated using outcome-wide multivariable regression adjustment (RA), propensity score matching (PSM), and instrumental variable (IV) analyses.

**Results:**

At baseline, 60% of respondents had a low SNI. Results from the RA and PSM models showed greatest concordance and that among women a diverse SN was positively associated with pulmonary outcomes and upper and lower body functions. Both men and women with a high SNI reported less limitations in performing activities of daily living (ADL) and instrumental ADL (IADL) tasks. A high SNI was negatively associated with C-reactive protein levels in women. The IV analyses yielded positive associations with cognitive functions for both men and women.

**Conclusions:**

Diverse SN confer a wide range of strong and heterogeneous long-term health effects, particularly for older women. In settings with limited formal welfare protection, intervening in the SN of older adults and safeguarding their access to diverse networks can be an investment in population health, with manifold implications for health and public policy.

## Background

Embeddedness in social networks has always been a defining characteristic of our human species. Social networks are known to influence people’s health above and beyond the influence of their individual attributes, and these relational structures have been recognized as strong determinants of health throughout the life course [1–3]. A large body of research has linked social networks to various physical and mental health outcomes [4–7], health behaviors [8], and longevity [2, 9]. The basic association between social networks and mortality has been demonstrated more than fourty years ago [10] and more recently the independent effect of social neworks on mortality has been established with a magnitude of this effect being comparable to well-established risk factors like smoking and obesity [11].

Research from high-income countries strongly suggests that social networks are instrumental for healthy aging by protecting against a range of negative health outcomes, including chronic non-communicable disease (NCD) morbidity [12–15], by slowing down or aiding recovery from aging-related impairments [16, 17], cognitive decline [18, 19], disability [20–23], and mortality [10, 11, 24]. Many NCD risk factors spread through social networks [25, 26], and studies have also suggested that social networks affect patterns of health care utilization, predict institutionalizations [27, 28], and influence NCD self-management among aging populations [29, 30]. However, the generalizability of these associations to aging populations in low- and middle-income countries (LMICs) is not well established [6].

Despite the general consensus that social networks have an effect on health, there is a lack of a clear theoretical understanding of *how* they affect health, i.e. the intervening mechanisms and pathways linking social networks to various health outcomes [4, 31]. Social networks are commonly viewed as multifactorial constructs that can be characterized by their structural and functional components at the individual, family, community or society levels [32]. Structural indicators are commonly quantitative in nature (e.g. network size), while functional aspects refers to the functions provided by a network (e.g. social support, access to resources). The conceptualization and operationalization of social network indicators varies largely between studies and behind each concept may be different mechanisms and pathways at work. Recognizing that social networks are embedded in and operate through various multilevel phenomena, Berkman has presented a conceptual model of how social networks impact health [4]. The framework embeds social networks into a larger cascading causal process in which upstream macro-level social-structural forces condition the structures and features of mezzo-level social networks, which in turn, provide opportunities for a set of micro-level psychosocial mechanisms to fall into motion. These downstream mechanisms operate through the provision of social support, social influence, social engagement, or by providing access to resources and material goods. Ultimately, this affects health through proximate pathways including direct physiological responses, psychological states and traits, and health behaviours.

In our study, we focus on social network diversity. Being a structural indicator, network diversity is defined as the number of different network domains in which respondents are actively embedded in [33]. Particularly for older adults, there are several advantages why more diverse social networks confer health benefits over and above other commonly used structural indicators such as network size. Network diversity is an indicator for heightened social integration and participation which has been associated with various mental [34] and physical health benefits [11] among aging populations. Greater diversity indicates the availability of more diverse types of support, which is particularly useful for aging populations with increasingly varying and enduring health and social needs [35]. And given the tendency for decreasing network size and the clustering of network losses among older adults (e.g. entering retirement, widowhood), greater diversity offers multiple opportunities to compensate for such structural network holes during the aging process. While those with diverse networks are likely to be embedded into a large-sized network, the reverse may not always hold and it is therefore crucial to more clearly outline the role of social network diversity in the aging process.

In many low- and middle-income countries (LMICs), due to various hindrances, including a lack of resources, data, and political will, epidemiological studies and thus policy responses on the health effects of social networks for particularly aging populations are still uncommon [6, 36]. Studies have shown that, specifically in LMIC settings, social networks function as important safety nets, especially for older adults [37–39]. Particularly, given the widespread non-availability of formal support structures in such settings, the provision of informal support from various sources may be more crucial for maintaining health and wellbeing as compared to high-income settings and thus, the importance of interpreting adult health outcomes in terms of social networks becomes critical. Health insecurity and limited social welfare protection in health stemming from weak and fragmented health care systems, lack of adequate health insurance schemes, and financial and geographical access barriers have become a major concern in many LMICs [40]. Cross-cultural research has shown that in many non-Western cultures social networks tend to be larger by means of extended family structures and strong community bonds, but studies investigating the role of network diversity remain scarce in such settings. Particularly, research on the health effect of network diversity among vulnerable populations such as older adults remains limited. With a traditional (and continuing) focus on children, mothers, infectious diseases, and nutrition, health care systems in LMICs are in many ways overburdened by population aging and its rising demands for care addressing NCDs, aging-related physical and cognitive decline, and the provision of long-term geriatric services. Especially due to the chronic incapacitating and disabling nature of NCDs, the growing numbers of older adults in LMICs rely on support from their informal social networks, that is, family, friends, or other informal groups, to enhance their chances of health and well-being. Thus, intervening in the social networks of older adults can be an investment in population health, with manifold implications for health and public policy. However, to formulate policies, one should know the degree to which social networks influence health, which aspects of health are affected, and which groups should be targeted.

### The Indonesian setting

Indonesia – the world’s fourth most populous country – represents a particularly interesting case for the study of social networks and adult health and aging. During the past decades, Indonesia’s population has aged, undergoing a swift demographic transition from high to low levels of both fertility and mortality. As a result, life expectancies have risen from 55 years in 1971 to 67 years for men and 72 years for women in 2017 [41–43]. Indonesia’s age structure has gradually transitioned towards higher age groups. In the 1970s, people over the age of 60 years made up 4.5% of the population, but in 2015 this figure had risen to 8.5%, and projections estimate that it will almost double to 16% by 2035 [44, 45]. Such a development will have far-reaching socio-economic implications and put pressure on the existing intergenerational support systems [41, 46, 47]. Predictions based on population censuses show clearly that many of the country’s older people will need more economic aid as their labour force participation declines, and social pensions are often not sufficient [43, 44]. Many will need to engage in income-generating activities past retirement age to meet their basic needs [48, 49]. Old-age care responsibilities traditionally lie with children [50], but there will also be a growing number of older couples with fewer (or increasingly no) children on whom they can depend [41, 51]. Additionally, Indonesia is facing the feminization of aging, with a larger number of women, many of them widows, living alone. These women will reach old age while facing a range of challenges imminent to their lower educational attainment and labour force participation [41, 42, 45, 48].

Intertwined with this demographic transition of Indonesia is the epidemiological transition towards a rising burden of NCDs. A rising prevalence from 48% in 1990 to 70% in 2010 shows that, over the past decades, NCDs have decisively replaced infectious diseases and malnutrition as the leading causes of death and disability in Indonesia [52, 53]. The latest data show that NCDs result in 73% of all deaths in Indonesia, identifying cardiovascular diseases (CVDs) (35%), cancers (12%), diabetes (6%), and chronic respiratory diseases (6%) as the leading causes of death [54]. The proportional mortality rates due to NCDs are especially high (84%) among adults over 50 years of age [55]. The risk of premature mortality due to NCDs increased from 23% in 2014 [56] to 26% (men 30%; women 23%) in 2018, with stroke and ischemic heart diseases being the main causes [54]. Aging-related disability likewise increased; specifically, the percentage of adults aged over 60 years who reported at least one disability increased from 11% in 2007 to 26% in 2018 [53, 57].

Scholars and policy makers are only beginning to recognize the health and social policy challenges of population aging in Indonesia. This development could generate an unprecedented increase in the number of patients in need of long-term chronic care and geriatric services [58] and present a major challenge to the country’s ambition to achieve universal health coverage (UHC) and health system equity [59–61]. Despite the importance of interpreting adult health outcomes in terms of social networks, particularly in LMICs, the empirical research on Indonesian populations is limited and policy attention is lagging [46, 62]. A systematic review synthesizing the evidence from Indonesia between the years 2000 and 2016 emphasized the need for more social network research involving health among aging populations [61]. Several qualitative and ethnographic studies have been conducted in East Java and West Sumatra, offering insights inter alia into issues like intergenerational family support systems [63], childlessness and old-age vulnerabilities [64], or care dependencies in relation to family network gaps [65, 66]. During the past years, few quantitative studies have emerged on related concepts, such as social capital (and healthy aging in seven eastern provinces) [67] and social engagement (and productive aging in ten rural villages) [68]. To the best of our knowledge, no epidemiological social network studies on aging populations are at present available for the larger Indonesian context. This study not only offers to fill this gap but also responds to calls (e.g. [4, 6, 69] to provide more evidence on the causal associations between social networks and health and particularly to strengthen the evidence base for certain sub-populations, such as older adults in LMICs.

### Novelties, rational and analytical hypotheses

Compared with other studies scrutinizing aspects of the social network–health relationship, we believe that this study offers two novelties. Firstly, it is, to the best of our knowledge, the first to adopt an outcome-wide epidemiological approach [70] to the investigation of the effects of social networks on health in general, and older adults’ health in Indonesia in particular. By utilizing this approach, one can see the effects of a specific exposure – in our case social network diversity – on multiple health outcomes simultaneously. As opposed to studies focusing on one exposure and one outcome at a time or exposure-wide epidemiological studies, the results of such an approach are particularly useful for decision makers when they need guidance on prioritizing certain public health recommendations over others. Secondly, we implemented a multi-method causal inference approach comparing the results from two methods that estimate causal effects by confounder-control approaches with an instrument-based approach [71]. With the premise that no single method is perfect or guarantees a true answer, employing different methods, each with different limitations, can give greater confidence to the validity of one’s conclusions. Additionally, by applying both confounder control and instrument-based approaches, we are able to draw conclusions both on individual and population level since these techniques produce effect estimates which apply to different populations [72, 73]. While the confounder-based approaches shed light on questions such as if greater social network diversity confers better health outcomes for individual respondents, the instrument-based approach is meant to inform decision-making on a population level, i.e. will an increase in network diversity among older adults at population level lead to bettter health outcomes? It has been widely acknowledged that such multiple-method analytical approaches are a key ingredient of any serious evaluation strategy [74, 75]. We believe that this study is the first attempt to apply a multi-method approach to the investigation of social networks’ health effects on older adults in a LMIC setting.

The aim of our study is to outline more clearly the role of social network diversity in the aging process in Indonesia. Our primary research question centres on whether and to what degree there is an association of social network diversity with adult health in Indonesia. To answer this, the present study takes an outcome-wide analytic approach [70] to examine prospectively the associations of social network diversity on a wider battery of adult health outcomes. To frame our outcome-wide approach, we apply Verbrugge and Jette’s sociomedical “disablement process” model [76]. In this model, age-related disability is generally regarded as the common endpoint being not only a function of preceding pathologies, impairments, and functional limitations but also the result of an adaptive process subject to various intra-individual, extra-individual, and risk factors. We focus on social networks as being one such factor affecting, for example speeding or slowing, the pathway from pathology to disability via impairments and functional limitations. In relation to this, we propose three broader sets of hypotheses.

First, based on the premise that gender is a crude indicator of the broader macro-sociocultural context of social interactions [4], we particularly aim to explore the differences in the health-protecting potential of women’s and men’s social networks. Second, given that the findings on the relationship between social networks and adult health have so far varied, with studies reporting positive, negative, or no associations [77, 78], we hypothesize that the dose–response relationship between social network diversity and health outcomes along the disablement process model is not linear, and we expect heterogeneous health effects, very likely varying by sex/gender. In this sense, there is further added value in applying an outcome-wide approach, because it has been noted that the application of an outcome-wide approach is particularly beneficial if the exposure does not equally affect all health outcomes in the same direction [70]. Third, with the disablement process model as the conceptual base for our analyses and the notion of social networks being a psychosocial determinant of health, we seek to clarify whether there is a gradient within the model from weaker associations between social networks and outcomes manifested on cellular or body system levels (i.e. pathologies and impairments) to larger effects on dimensions relating to the person or the person’s relation to society (i.e. functional limitations and disabilities). This hypothesis is based on other studies that have investigated the mediating role of psychosocial determinants, such as social integration, social support, or loneliness, within the disablement process [79–81]. We specifically chose to focus on network diversity, that is, the number of different network types in which a respondent is embedded based on the long-standing notion that more diverse networks may produce health benefits over and above the crude network size [10, 33, 82]. Particularly for aging populations, networks with greater diversity represent opportunities for various types of support, which may not be available for older adults with large but less diverse networks.

## Methods

### Data source: the Indonesian Family Life Survey

The data for this analysis were sourced from the fourth and fifth waves of the Indonesian Family Life Survey (IFLS), hereafter referred to as IFLS-4 and IFLS-5 [83, 84]. The IFLS is a continuing longitudinal socioeconomic and health survey that started in 1993, making it the longest panel study outside OECD territory [85]. The IFLS is also one of few surveys in an LMIC setting that has implemented the large-scale collection of biomarkers using dried blood spots (DBS) testing [86]. The IFLS-1 baseline sample from 1993 contains 30,000 individuals from 7224 randomly selected households, representing about 83% of the Indonesian population living in 13 provinces. Almost 88% of the original IFLS-1 dynasty has been interviewed in all 5 waves. For this study, we use IFLS-4 and IFLS-5, which were fielded in 2007–08 and 2014–15 on the 1993 households and their split-offs. The dynasty recontact rate in IFLS-4 and IFLS-5 was 94 and 92%, respectively. The recontact rate of IFLS-5 with IFLS-4 households was 91%. For those who had died since the completion of IFLS-4, interviews with a knowledgeable proxy were conducted. Further details of the IFLS’s sampling scheme and recontact protocols are available elsewhere [83, 84, 87]. The IFLS data are open for public use upon registration on the website of the RAND Corporation (www.rand.org/labor/FLS/IFLS). RAND, Survey Meter, and Gadjah Mada University, which undertook IFLS-4 and IFLS-5, obtained ethical approval.

### Measures

(i) *Exposure: Social Network Index*

The primary exposure variable is the diversity of each respondents’ social network at the baseline. This was measured through a composite measure, a social network index (SNI), combining information about the household size together with the range of social ties with whom respondents had active contact across six different types of role relationship, including a spouse, parents, children, siblings, neighbours, and members of groups without and with religious affiliation. All these variables were sourced from IFLS-4. For the calculation of the SNI, we followed with few modifications the procedures described by Cohen and colleagues [33]. One point was assigned to each type of relationship, and we gave equal weight to intimate, kin and non-kin relationships. Therefore, a respondent living in a household with more than four persons (the Indonesian national average household size), who is married, whose parents (if alive; or parents-in-law) co-reside in the same household, who provides and/or receives instrumental or financial support to/from siblings (or siblings-in-law) and children (biological, not co-resident), and who participates in social and religious activities monthly received the full score of seven points and thus was classified as a respondent with a diverse social network. The IFLS is not particularly geared towards measuring social networks or their diversity. Therefore, we needed to employ a variable which measured reciprocal instrumental and economic support to/from siblings and children as a proxy for indcating the presence of such kin networks. This is a strength of our index, as we can control to some extent for the correlation between structural and functional characteristics of social networks, because the common assumption that more diverse networks should automatically be associated with increased receipt of support may not always be true. The Cronbach alpha of the SNI was 0.718. Based on the data-driven median split, we then dichotomized the respondents into those with low social network diversity (SNI score: 0–4 points) and those with a diverse social network (score: 5–7 points). Most respondents (84.7%, *n* = 2591) had no missing data for any of the 7 indicators used to compute the SNI; the remaining 469 had two or less missing responses but could still be assigned a SNI. The risk for exposure-dependent misclassification of this approach was low and affected < 1% of respondents.

Approximately 1% of the respondents reported null network diversity (SNI = 0: *n* = 2; SNI = 1: *n* = 31), which further confirms that our study is primarily examining the effect of network diversity and not related concepts, such as social isolation, on adult health outcomes. Further details of the derivation of the analytical sample size are provided below.

(ii) *Outcome battery*

In our study, a wider battery of 19 health outcomes measured in 2014/15 were considered. Guided by the disablement process model [76], we grouped these outcomes into four interrelated components: eight pathologies, five impairments, four functional limitations, and two disabilities. Table 1 presents a summary of the different outcomes. Further details are available in the IFLS-5 field report [84] and the DBS data user guide [86]. Biomarker data for C-reactive protein (CRP) and glycated haemoglobin (HbA1c) levels are only available for a random sub-sample of respondents (CRP: *n* = 1913, 62.5%; HbA1c: *n* = 1887, 61.7%). For details, see the annotations in Table 1.

(iii) *Covariates*

**Table 1:** Overview and assessment of the outcome battery

**Outcome**	**Type of measurement**	**Response scale**
**I) Pathologies**		
1. Non-communicable disease (NCD) morbidity	Self-reported physician diagnosis^1^ of any one condition listed #2-#7; “Has a doctor ever told you that you had […]?”	0 = yes; 1 = no
2. Asthma	Self-reported physician diagnosis^1^	0 = yes; 1 = no
3. Other chronic lung diseases	Self-reported physician diagnosis^1^	0 = yes; 1 = no
4. Cancer or malignant tumours	Self-reported physician diagnosis^1^	0 = yes; 1 = no
5. Diabetes or high blood sugar	Self-reported physician diagnosis^1^	0 = yes; 1 = no
6. Cardiovascular diseases (heart attacks, coronary heart diseases, angina, or other heart problems)	Self-reported physician diagnosis^1^	0 = yes; 1 = no
7. Stroke	Self-reported physician diagnosis^1^	0 = yes; 1 = no
8. Prediabetes or diabetes based on glycosylated haemoglobin (HbA1c) levels^2^	Biomarker; Dried blood spots (DBS) based assays taken from trained IFLS interviewers to measure glucose metabolism.	Continuous variable (range: 3.5–12.8%); Binary variable: 0 = yes (diabetes or prediabetes, > 5.7%); 1 = no (normal range < 5.7%)^3^
**II) Impairments**		
*Cardiovascular impairments:* 9. Hypertension	Three measures of systolic and diastolic blood pressure (BP) in mmHg on alternate arms (starting left) by trained IFLS interviewers using an Omron meter (HEM-7203) and self-reported use of antihypertensive medication.	0 = yes (hypertensive; defined as mean systolic BP ≥140 mmHg and/or mean DBP ≥90 mmHg and/or current use of antihypertensive medication); 1 = no (normotensive)^4^
*Immunological impairments:* 10. Chronic inflammation based on C-reactive protein (CRP) levels^2^	Biomarker; CRP (plasma equivalent) concentrations from finger-prick DBS specimens measured using validated enzyme-linked immunosorbent assay (ELISA) method.	Continuous variable (range: 0.01–58.95 mg/l); Binary variable: 0 = yes (> 1.0 mg/l); 1 = no (normal range < 1.0 mg/l)^5^
*Muscoskeletal impairments:* 11. Mean hand grip strengths	Physical performance test; Hand grip strengths was measured by a trained IFLS interviewer using a Baseline Smedley Spring type dynamometer (daily calibration). Respondents were asked to squeeze the dynamometer in each hand twice beginning with the dominant hand. Two measurements per hand were recorded including information on any recent surgery, swelling, inflammation, severe pain, or injury on one or both hands and recording of dominant hand.	Continuous variable (mean of four measurements, range: 0–47.75 kg)
12. Arthritis and/or rheumatism	Self-reported physician diagnosis^1^	0 = yes; 1 = no
*Sensory impairments:* 13. Hearing and/or vision problems	Self-reported physician diagnosis^1^	0 = yes; 1 = no
**III) Functional limitations**		
*Physical functional limitations* 14. Upper-body functional limitations (UBFL)^6^	Self-reported physical functioning measures; including show cards; Question: “If you had […], could you do it?” 1) to carry a heavy load (like a pail of water) for 20 m 2) to draw a pail of water from a well 3) to sweep the house / floor / yard	0 = yes (includes responses “with difficulty” and “unable to do it”); 1 = no (“easily”)
15. Lower-body functional limitations (LBFL)^7^	Self-reported physical functioning measures; including show cards; Question: “If you had […], could you do it?” 1) to walk 1 km 2) to bow, squat, or kneel 3) to stand up from sitting on the floor without help	0 = yes (includes responses “with difficulty” and “unable to do it”); 1 = no (“easily”)
*Cognitive functional limitations* 16. Episodic memory score	Cognitive performance test; Immediate and delayed word recall of ten nouns. These were read out slowly and the respondent was asked to repeat the list immediately and again after 4 to 5 min. Questionnaire contained four lists of each ten words which were randomized across individuals within a household.	Continuous variable (mean number of words correctly recalled for both immediate and delayed response; range: 0–8.5 words)
17. Visuospatial ability score	Cognitive performance test; IFLS uses an abridged version of the Raven’s Progressive Matrices^8^, a non-verbal self-paced test in which each item contained a pattern with a missing part. The respondent had to infer the rules underlying the pattern and apply these rules to discover which of the answer options provides the correct completion for a total of eight items.	Continuous variable (one score point per correct answer; range: 0–8 points)
**IV) Disabilities**		
18. Activities of daily living (ADLs) limitations^9^	Self-reported physical functioning measures for five basic tasks of everyday life; including show cards; Question: “If you had […], could you do it?” (1) to dress without help (2) to bathe (3) to get out of bed (4) to eat (eating food by oneself when it is ready) (5) to control urination or defecation	Continuous variable (range 5–25); Binary variable: 0 = yes (includes responses “with difficulty”, “can do with help” and “unable to do it”); 1 = no (“easily”)
19. Instrumental activities of daily living (IADLs) limitations^10^	Self-reported ability to perform IADLs items; including show cards); Question: “If you had […], could you do it?” (1) to shop for personal needs (2) to prepare hot meals (prepare ingredients, cooking, and serving food) (3) to take medicine (right portion at right time) (4) to do household chores (house cleaning, doing dishes, making the bed, and arranging the house) (5) to shop for groceries (deciding what to buy and pay for it) (6) to manage your money (paying your bills, keeping track of expenses, or managing assets)	Continuous variable (range 6–30); Binary variable: 0 = yes (includes responses “with difficulty”, “can do with help” and “unable to do it”); 1 = no (“easily”)

Annotations Table 1:

For more details see Strauss J, Witoelar F, Sikoki B. The Fifth Wave of the Indonesia Family Life Survey (IFLS5): Overview and Field Report. Santa Monica: RAND, 2016 and IFLS questionnaires available at https://www.rand.org/well-being/social-and-behavioral-policy/data/FLS/IFLS.html

1) Includes diagnoses by paramedics, nurses and midwifes

2) HbA1c and CRP values are only available for a sub-sample in IFLS-5. DBS for CRP assays were introduced in IFLS-4 for a random sample of IFLS-1 dynasty households (=9944 respondents above age 1). In IFLS-5, the target sample for repeated CRP assays and (newly introduced) HbA1c was the subset of respondents who had DBS taken in IFLS-4. There are 7579 observations with CRP data and 7524 observations with HbA1c in wave 5. Further details on sampling for the DBS and sampling weights are available in Herningtyas EH, Hu P, Edenfield M, Strauss J, Crimmins E, Witoelar F, et al. IFLS Wave 5 Dried Blood Spot Data User Guide. Santa Monica: RAND, 2018. In our analyses, we have CRP data for 1913 (35%) and HbA1C data for 1887 (34%) respondents

3) Cut-offs based on The International Expert Committee. Report on the role of the A1C assay diagnosis of diabetes. Diabetes Care. 2009 32(7):1327–34

4) Respondents with controlled hypertension (*n* = 62), uncontrolled hypertension (*n* = 2262) and hypertension without treatment (*n* = 33) were classified as hypertensive. Hypertension definition adapted from WHO Expert Committee on Hypertension Control. Hypertension control. Geneva: World Health Organization, 1996

5) Cut-offs are based on Speidl WS, Zeiner A, Nikfardjam M, Geppert A, Jordanova N, Niessner A, et al. An increase of C-reactive protein is associated with enhanced activation of endogenous fibrinolysis at baseline but an impaired endothelial fibrinolytic response after venous occlusion. Journal of the American College of Cardiology. 2005 45 (1):30–4

6) Cronbach’s alpha for three UBFL items = 0.7863

7) Cronbach’s alpha for three LBFL items = 0.7306

8) Raven J. The Raven’s progressive matrices: change and stability over culture and time. Cogn Psychol. 2000 41 (1):1–48

9) Cronbach’s alpha for five ADL items is 0.8319; ADL items adapted from Katz S. Assessing self-maintenance: activities of daily living, mobility, and instrumental activities of daily living. J Am Geriatr Soc. 1983 31 (12):721–7

10) Cronbach’s alpha for six IADL items is 0.9043; IADL items adapted from Lawton, M.P., & Brody, E.M. (1969). Assessment of older people: Self-maintaining and instrumental activities of daily living. The Gerontologist, 9 (3), 179–186

Following Caliendo and Kopeinig, we selected a rich set of 15 covariates that satisfied the unconfoundedness assumption necessary for the later estimation of treatment effects [88]. Guided by the disablement process model [76] and a systematic review providing information on the social determinants of adult health in Indonesia [61], the following individual and household sociodemographic covariates were chosen: (i) sex/gender (men, women), (ii) age (50–57 y, > 57 y), (iii) educational attainment (no schooling, elementary school, high school, university), (iv) quartiles of monthly household per capita expenditure (PCE) (the first quartile contains the poorest 25%), (v) residential stability (moved once or more since year 2000 vs no change in residence), and (vi) area of residence (rural, urban). We decided to group people into two age groups dichotomized at the age of 57, the 2014 average Indonesian retirement age. The PCE was pre-calculated by the IFLS from the monthly total household expenditures for food and non-food consumption and expenditures including purchased goods, services, and durables as well as housing and education-related expenditures in Indonesian Rupiah divided by the number of household members [89]. We used the household PCE as a proxy for income and living standard. Being a potential barrier to social network development, we decided to include a variable indicating residential instability. The information was drawn from IFLS-4 but refers to the time window between IFLS-3 (fielded in 2000) and the 8 years leading up to the fielding of IFLS-4. Besides these, we included the following health behaviours and biological risk factors: (vii) respondents’ physical activity level (vigorous, moderate, less), (viii) smoking status (no, yes), and (ix) body mass index (BMI) (normal weight, underweight, overweight, obese). Other health-related covariates included: (x) overall self-rated health (bad, good), (xi) self-reported depressive symptoms (yes, no), (xii) general health check-up in the past 5 years (no, yes; information taken from IFLS-5), and (xiii) health insurance coverage (no, yes). For the BMI, we used the anthropometric cut-off points suggested for Asian populations [90] (see the annotations in Table 2 for the exact cut-off values). To rule out reverse causation, we controlled for the presence of (xiv) NCDs and (xv) disability at the baseline. All 15 covariates and the SNI with baseline descriptive statistics are presented in Table 2.

**Table 2:** Baseline characteristics of respondents in IFLS-4 (2007/08), stratified by sex/gender

**Covariates**	**Total** (*N* = 3060)	**Men** (*n* = 1477, 48%)	**Women** (*n* = 1583, 52%)	***p-value***
	n (%) or mean ± SD	n (%) or mean ± SD	n (%) or mean ± SD	
**Social network index**				
SNI score	4.15 (1.14)	4.18 (1.02)	4.12 (1.23)	0.147
Low SNI	1840 (60)	890 (60)	950 (60)	0.890
High SNI	1220 (40)	587 (40)	633 (40)	
**Age group**				
50–57 years	1736 (57)	813 (55)	923 (58)	0.069
57+ years	1324 (43)	664 (45)	660 (42)	
**Education**				
No schooling	553 (18)	145 (10)	408 (26)	< 0.001
Elementary school	1706 (56)	847 (57)	859 (54)	
High school	631 (21)	369 (25)	262 (17)	
University	170 (6)	116 (8)	54 (3)	
**Household PCE**^**1**^				
First (poorest)	735 (24)	363 (25)	372 (23)	0.336
Second	783 (26)	388 (26)	395 (25)	
Third	770 (25)	350 (24)	420 (27)	
Fourth (richest)	772 (25)	376 (25)	396 (25)	
**Living area**				
Rural	1611 (53)	812 (55)	799 (50)	0.013
Urban	1449 (47)	665 (45)	784 (50)	
**Residential stability**				
Moved once or more since 2000	280 (9)	167 (11)	113 (7)	< 0.001
Did not move since year 2000	2780 (91)	1310 (89)	1470 (93)	
**Smoking**				
Yes	1259 (41)	1132 (77)	127 (8)	< 0.001
No	1801 (59)	345 (23)	1456 (92)	
**Body mass index**^**2**^				
Obese	385 (13)	117 (8)	268 (17)	< 0.001
Overweight	1010 (33)	429 (29)	581 (37)	
Underweight	1211 (40)	698 (47)	513 (32)	
Normal weight	454 (15)	233 (16)	221 (14)	
**Physical activity level**				
Less active	553 (18)	236 (16)	317 (20)	< 0.001
Moderately active	1503 (49)	554 (38)	949 (60)	
Vigorously active	1004 (33)	687 (47)	317 (20)	
**Self-rated health**				
Bad	548 (18)	218 (15)	330 (21)	< 0.001
Good	2512 (82)	1259 (85)	1253 (79)	
**Self-reported depressive symptoms**				
Yes	1064 (35)	528 (36)	536 (34)	
No	1996 (65)	949 (64)	1047 (66)	0.273
**Health check-up in the past 5 years**^**3**^				
No	2776 (91)	1329 (90)	1447 (91)	0.173
Yes	284 (9)	148 (10)	136 (9)	
**Health insurance coverage**^**4**^				
No	2177 (71)	1033 (70)	1144 (72)	0.155
Yes	883 (29)	444 (30)	439 (28)	
**NCD morbidity**^**5**^				
Multiple NCDs	25 (1)	14 (1)	11 (1)	0.262
Single NCD	273 (9)	121 (8)	152 (10)	
None	2762 (90)	1342 (91)	1420 (89)	
**Disability**^**6**^				
ADL and IADL	256 (8)	54 (4)	202 (13)	< 0.001
ADL or IADL	625 (21)	152 (10)	473 (30)	
No disability	2179 (71)	1271 (86)	908 (57)	

Annotations Table 2:

1) Monthly household per capita expenditure in Indonesian rupiah (IDR); the mean household PCE amount equals ca. USD 59 (IDR 1 = USD 0.0001107690 as of midyear 2007)

2) Based on the BMI cut-off points suggested for Asian populations: < 18.48 = underweight; 18.50–22.99 = normal weight; 23.00–27.49 = overweight; > 27.50 = obese. Source: WHO Expert Consultation. Appropriate body-mass index for Asian populations and its implications for policy and intervention strategies. Lancet. 2004 363(9403):157–63

3) Information taken from IFLS-5 (2014/15)

4) Includes health insurance programmes from ASKES, ASTEK/Jamsostek, employer provided medical reimbursement, employer provided clinic, private health insurance, savings-related insurance, JAMKESMAS, JAMKESDA, JAMKESSOS, JAMPERSAL, or ASURANSI MANDIRI

5) The crude measure of baseline NCD morbidity includes self-reported physician diagnoses of asthma, other chronic lung diseases, cancer or malignant tumours, diabetes or high blood sugar, heart attacks, coronary heart diseases, angina, other heart problems, and strokes

6) In the IFLS questionnaires, three IADL items across wave 4 and wave 5 were not identical. The following items have been compared in IFLS-4 and IFLS-5, respectively: (i) performing household chores vs sweeping the floor; (ii) shopping for groceries vs visiting a friend in the same village; and (iii) managing money vs taking a trip out of town

### Statistical analysis

The distributions of the 2007/08 baseline characteristics and the prevalence of health outcomes in the 2014/15 follow-up are presented in Tables 2 and 3. Continuous variables are presented as the mean with standard deviation (SD) and compared with Fisher’s t-tests. Numbers (n) and proportions (%) are presented for categorical variables, which are compared with Pearson’s chi-squared tests. A *p*-value below 0.05 signifies statistical significance. Following Pearl’s transdisciplinary causal inference framework [71, 91], three methods were used to estimate the effects of social network diversity on adult health outcomes: two confounder-control approaches and one instrument-based approach. In the first, we used regression adjustment (RA) models to break the association of confounders with the outcome and propensity score matching (PSM) models to break the association of the confounder with the exposure. In the later, similar to a natural experiment approach, we address potentially unmeasured confounding and measurement error of the exposure-outcome association by leveraging an exogenous source of variation in form of an instrumental variable. All the models are based on complete case analyses.

**Table 3:** Distribution of health outcomes according to the baseline SNI, stratified by sex/gender

**Health outcomes**		**Total** (n = 3060)	**Mean** **social networkindex (SNI)**: 4.2 (1.1)	**Low SNI** (n = 1840, 61%)		**High SNI** (n = 1220, 39%)		***p-value***
				Men (n = 890, 29%)	Women (n = 950, 31%)	Men (n = 587, 19%)	Women (633 (21%)	
		n (%) or mean (SD)	Mean (SD)	n (%) or mean (SD)	n (%) or mean (SD)	n (%) or mean (SD)	n (%) or mean (SD)	
**Pathologies**	**NCD morbidity**							
	Yes	497 (16)	4.19 (1.13)	134 (47)	152 (53)	107 (51)	104 (49)	
	No	2563 (84)	4.14 (1.14)	756 (49)	790 (51)	480 (47)	537 (53)	0.353
	**Asthma**							
	Yes	95 (3)	3.97 (1.02)	22 (35)	41 (65)	20 (63)	12 (37)	
	No	2965 (97)	4.16 (1.14)	868 (49)	909 (51)*	567 (48)	621 (52)	0.211
	**Other chronic lung diseases**							
	Yes	67 (2)	3.74 (1.06)	26 (51)	25 (49)	10 (63)	6 (38)	
	No	2993 (98)	4.16 (1.14)*	864 (48)	925 (52)	577 (48)	627 (52)	0.007
	**Cancer or malignant tumours**							
	Yes	21 (1)	4.9 (0.89)	3 (43)	4 (57)	6 (43)	8 (57)	
	No	3039 (99)	4.15 (1.14)*	887 (48)	938 (52)	581 (48)	633 (52)	0.012
	**Diabetes or high blood sugar**							
	Yes	205 (7)	4.32 (1.17)	52 (49)	55 (51)	51 (52)	47 (48)	
	No	2855 (93)	4.14 (1.14)*	838 (48)	895 (52)	536 (48)	586 (52)	0.016
	**Cardiovascular diseases**							
	Yes	132 (4)	4.19 (1.17)	42 (51)	41 (49)	24 (49)	25 (51)	
	No	2928 (96)	4.15 (1.13)	848 (48)	909 (52)	563 (48)	608 (52)	0.510
	**Stroke**							
	Yes	71 (2)	4.27 (1.12)	23 (61)	15 (39)	16 (48)	17 (52)	
	No	2989 (98)	4.15 (1.14)	867 (48)	935 (52)	571 (48)	616 (52)	0.250
	**HbA1c levels**							
	Mean level (%)	5.89 (1.23)	–	5.88 (1.19)	5.83 (1.12)	5.94 (1.21)	5.94 (1.43)	0.201
	Prediabetes, diabetes level	670 (46)	4.18 (1.14)	189 (48)	205 (52)	136 (49)	140 (51)	
	Normal levels ***[not measured: 1618 (53)]***	772 (54)	4.16 (1.14)	215 (47)	245 (53)	141 (45)	171 (55)	0.764
**Impairments**	**Hypertension**							
	Hypertensive	1796 (59)	4.11 (1.15)	487 (44)	612 (56)	302 (43)	395 (57)	
	Normotensive	1264 (41)	4.21 (1.12)*	403 (54)	338 (46)*	285 (54)	238 (46)*	0.153
	**CRP levels**							
	Mean level (mg/l)	2.21 (4.22)	–	2.30 (5.44)	2.10 (3.67)	1.95 (3.35)	2.48 (3.82)	0.854
	Medium or high risk level	658 (45)	4.24 (1.16)	162 (43)	211 (57)	122 (43)	163 (57)	
	Low risk level ***(not measured: 1603 (52))***	799 (55)	4.12 (1.12)*	245 (50)	244 (50)*	158 (51)	152 (49)*	0.081
	**Grip strengths**							
	Mean strengths (kg)	21.05 (7.53)	–	25.75 (6.59)	16.02 (4.80)*	26.63 (6.47)	16.81 (4.87)*	0.004
	**Arthritis and/or rheumatism**							
	Yes	402 (13)	4.17 (1.11)	83 (35)	156 (65)	55 (34)	108 (66)	
	No	2658 (87)	4.14 (1.14)	807 (50)	794 (50)*	532 (50)	525 (50)*	0.766
	**Sensory impairments**							
	Yes	345 (11)	4.31 (1.14)	84 (45)	104 (55)	66 (42)	91 (58)	
	No	2715 (89)	4.13 (1.13)*	806 (49)	846 (51)	521 (49)	542 (51)	0.023
**Functional limitations**	**Upper-body functional limitations**							
	Yes	1043 (34)	4.03 (1.17)	219 (33)	454 (67)	121 (33)	249 (67)	< 0.001
	No	2017 (66)	4.22 (1.12)*	671 (58)	496 (42)*	466 (55)	384 (45)*	
	**Lower-body functional limitations**							
	Yes	1140 (37)	4.06 (1.15)	246 (35)	467 (66)	168 (39)	259 (61)	
	No	1920 (63)	4.21 (1.13)*	644 (57)	483 (43)*	419 (53)	374 (47)*	0.036
	**Episodic memory score**							
	Mean number of words recalled (range: 0–10)	3.08 (1.52)	–	3.06 (1.47)	2.83 (1.53)*	3.35 (1.54)	3.24 (1.48)	< 0.001
	**Visuospatial ability**							
	Mean score (range: 0–8)	3.06 (2.01)	–	3.17 (2.03)	2.76 (1.85)*	3.53 (2.11)	2.93 (2.00)*	0.005
**Disabilities**	**ADL limitations**							
	Mean score (range: 5–25)	5.37 (1.29)	–	5.34 (1.36)	5.56 (1.62)*	5.24 (0.87)	5.28 (0.88)	< 0.001
	Any ADL limitation(s)	377 (12)	3.95 (1.15)	92 (36)	161 (64)	53 (43)	71 (57)	
	No ADL limitations	2683 (88)	4.18 (1.13)*	798 (50)	789 (50)*	534 (49)	562 (51)	0.003
	**IADL limitations**							
	Mean score (range: 6–30)	7.50 (3.63)	–	7.83 (3.99)	7.75 (4.03)*	7.25 (3.11)	6.91 (2.72)*	< 0.001
	Any IADL limitation(s)	715 (23)	4.01 (1.12)	239 (50)	235 (50)	131 (54)	110 (46)	
	No IADL limitations	2345 (77)	4.20 (1.14)*	651 (48)	715 (52)	456 (47)	523 (53)*	< 0.001

Annotations Table 3:

1) *(asterix) indicates a statistical difference (p < 0.05) of the mean SNI score between respondents with good vs bad health outcomes (column 3) and between men and women within each SNI group (columns 5 and 7)

2) The p-value (column 8) indicates a statistical difference between respondents with a low vs a high SNI

Note: *p*-values obtained from chi-squared tests for proportions and t-tests for means. Source: IFLS

Multivariable linear and logistic RA models were used to estimate the independent association between the SNI and any health outcome for the total analytical sample and then for men and women separately, controlling for the 15 covariates described above. Variables associated with outcomes at *p* < 0.05 were subsequently included in the multivariable regression models. Prior to running the final models, we checked for potential multicollinearity between variables with bivariate correlation and variance inflation factor (VIF) tests, and none of these exceeded a critical value (mean VIF 1.36).

Since the SNI was not randomly assigned to this population, we performed confirmatory PSM analyses following the conventional RA models. PSM is a method that allows the use of observational data to estimate treatment effects and make causal inferences based on counterfactuals [92]. It is a multivariable scoring method that collapses the predictors of a treatment into a single value that represents the probability (i.e. propensity) of being treated, conditional on all the observed covariates. Matching based on the PS produces samples with the same distribution of covariates in treated and untreated subjects. It should be noted that, in this study, we used observational data and thus refer to the SNI as an exposure, not a treatment. We followed the steps described by Caliendo and Kopeinig to estimate the PS using a logit model and to calculate the average treatment effect (ATE) [88]. We used nearest-neighbour matching without replacement with a ratio of 1:2 (for the total sample analyses; 1:1 for the sex/gender-stratified models) and a 0.25 standard deviation caliper width to match respondents with a high SNI (5–7 points) with ones with a low SNI (0–4 points) [93, 94]. We performed PSM to isolate the effect of the exposure, a high SNI, above and beyond respondents’ individual characteristics, because the method allowed us to estimate the effect of having a diverse social network on the subsequent health status in a non-exposed sample (with a low SNI), if that same sample would have had a high SNI. Prior to running the PSM models, we inspected balance plots of the distribution of the propensity scores before and after matching. All the plots suggested a good support scenario; that is, they showed substantial overlap of the PS for the exposed sample and the controls after matching (Appendix 1).

Lastly, we performed an IV analysis. As opposed to traditional risk adjustment methods that rely on observable measures (such as RA or PSM), an IV factor in unmeasured or unobserved factors is a potential source of confounding [95]. In our study, residential stability, the length of exposure to an ecological setting, was our instrument of choice. This choice was based on our review of the existing literature backing up the association between residential stability and social networks (e.g. [96, 97]), and we created this variable from a question in IFLS-4 inquiring “How many times did you move since the interview in 2000 [i.e. IFLS-3] between villages and stayed for six months or more?” After confirming the validity and strengths of our chosen IV, we performed a two-step IV regression analysis in which we regressed the coefficients of poor health outcomes on the instrumental probability of having a diverse social network to determine whether social networks were associated with health outcomes through networks’ relationship with residential stability. After the IV model fit, we tested whether residential stability qualified as an endogenous and strong instrumental variable. Both the Durbin (*p* = 0.049) and the Wu–Hauser (*p* = 0.045) statistics confirmed the endogeneity of our IV. The F-test also confirmed the overall strengths of the instrument (F = 19.58, *p* = 0.002). All the IV models were adjusted for the same covariates as the RA and PSM models.

For all three methods, we applied an outcome-wide epidemiological approach in which a single exposure (i.e. SNI) is examined and its effects on multiple outcomes are considered simultaneously [70]. We used longitudinal panel data covering eight years in order to investigate the effect of the baseline SNI on 19 outcomes along the disablement process over time. All the analyses were performed using Stata/SE V.14.2 (StataCorp, College Station, Texas, USA). We used Stata’s *regress and logit* functions, the *teffects psmatch* and *psmatch2* packages, and *ivregress* for the RA, PSM, and IV analyses, respectively*.* The coefficient plots were produced with *coefplot* [98].

## Results

In this study, we restricted the analyses to respondents who were aged 50 years or older at the time when IFLS-4 was fielded and who were subsequently interviewed for IFLS-5 (*n* = 8049; =13% of the panel (*N* = 63,136)). Complete social network data were available for 5521 respondents (8% of the panel). The final analytic sample consisted of 3060 respondents. The analytic sample size for the biomarkers (CRP, HbA1c) was smaller. For details, see the annotations in Table 1.

**Fig. 1:**
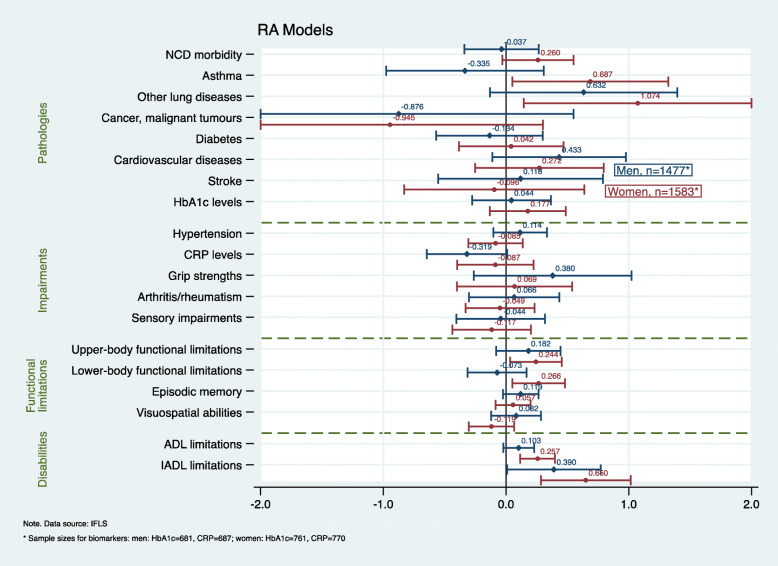
Results from the multivariable regression adjustment (RA) models for men (blue/diamond) and women (red/circle). Annotation Figure 1: The following confidence intervals (CIs) were truncated to a − 2 to 2 interval: lung diseases (women) 95% CI (0.1452047–2.002185); cancer (men) 95% CI (−2.302062–0.5509206); and cancer (women) 95% CI (− 2.191686–0.3012024). All the models were controlled for age, education, household per capita expenditure, residential stability, area of residence, physical activity, smoking, BMI, SRH, depression, health check-up in the past 5 years, health insurance coverage, baseline NCDs, and disability

**Fig. 2:**
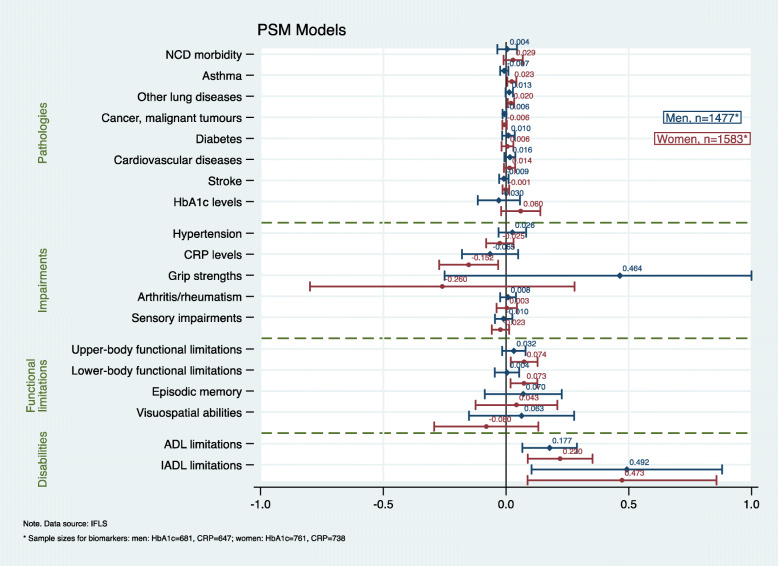
Results from the propensity score matching (PSM) models for men (blue/diamond) and women (red/circle). Annotation Figure 2: The following confidence interval (CI) was truncated to a − 1 to 1 interval: grip strengths (men) 95% CI (− 0.2499284–1.17726). All the models were matched on baseline age, education, household per capita expenditure, residential stability, area of residence, physical activity, smoking, BMI, SRH, depression, health check-up in the past 5 years, health insurance coverage, NCDs, and disability

**Fig. 3:**
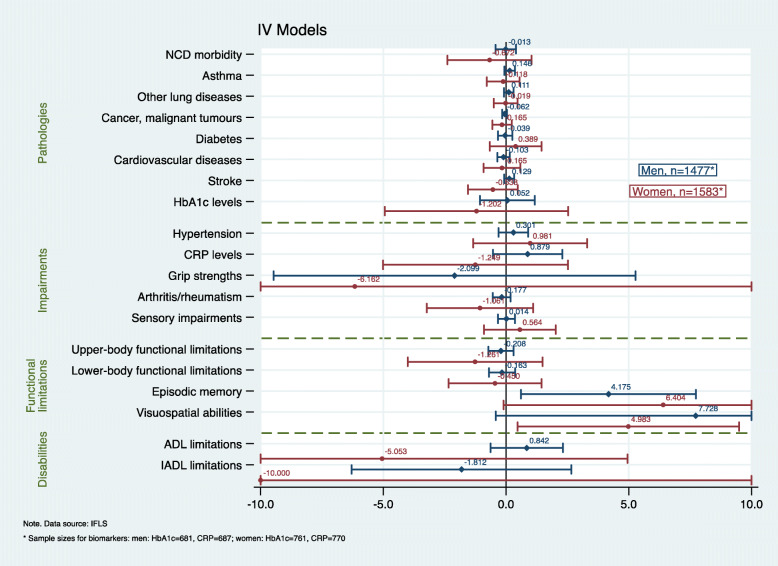
Results from the instrumental variable (IV) analysis models for men (blue/diamond) and women (red/circle). Annotation Figure 3: The following confidence intervals (CIs) were truncated to a − 10 to 10 interval: grip strengths (women) 95% CI (− 24.97352–12.64874); episodic memory (women) 95% CI (− 0.1034597–12.91134); visuospatial abilities (men) 95% CI (− 0.4174638–15.87406); ADLs (women) 95% CI (− 15.05158–4.946002); and IADLs (women): − 13.60297 (− 40.19411–12.98818). All the models were controlled for age, education, household per capita expenditure, area of residence, physical activity, smoking, BMI, SRH, depression, health check-up in the past 5 years, health insurance coverage, baseline NCDs, and disability; IV = residential stability (tests of endogeneity: Durbin (*p* = 0.049), Wu–Hauser (*p* = 0.045); instrument strengths F = 19.58 (*p* = 0.002)

In the full analytic sample, respondents predominantly reported a low SNI (60%), and there was no statistical difference in the mean SNI score between men and women (*p* = 0.147). A slightly higher percentage of respondents were women (52%), and, at the study baseline, the mean age was 58 years (SD 6.67, range 50–87 years). Men were more likely to reside in rural areas (55 vs 50%, *p* = 0.013) but also changed their place of residence more often than women (11% vs 7%, *p* < 0.001). Furthermore, the educational profiles, baseline SRH and disability, and health behaviours between men and women varied significantly (all *p*-values < 0.001). Further details about respondents’ baseline characteristics are shown in Table 2. Respondents’ health profile at follow-up according to their baseline SNI is presented in Table 3. The results from the RA, PSM, and IV models for men and women are displayed in the coefficient plots in Figs. 1, 2 and 3 (the results for the total sample are available from Appendices 2 and 3).

Overall, for the total sample, the RA models yielded the largest number of significant results (for 6/19 outcomes), followed by the PSM (5/19) and IV models (2/19). We found the highest level of concordance between the results from the RA and PSM models in both the total sample analyses (agreement on 4 significant results and 12 non-statistically significant effects, 84% concordance) and the sex/gender-stratified models (men: agreement on 1 significant and 17 insignificant results, 95% concordance; women: 6 significant and 12 insignificant results, 95% concordance). There was no concordance between the IV models and the RA or PSM models. Our results showed that, among women, a strong SNI subsequently affected all the dimensions of the disablement process, while, among men, its effects were restricted to the functional limitations and disability dimensions. Further, the effect sizes were generally larger and more heterogeneous among women than among men.

With regard to pathologies, we found that, among women, pulmonary diseases were associated with the baseline SNI. The RA models showed significant positive associations with self-reported asthma (coef.: 0.687, *p* = 0.018) and other chronic lung diseases (coef.: 1.074, *p* = 0.009). The application of PSM likewise confirmed that the propensity for exposure to a high SNI had a subsequent effect on asthma (ATE: 0.023, *p* = 0.002) and other chronic lung diseases (ATE: 0.020, *p* < 0.001).

The only statistically significant results for impairments were derived from the PSM models among the female sample. We found a significant negative association between social network diversity and immunological impairments measured by categorized (medium/high vs low) CRP levels. Elevated levels of CRP above 1 mg/l were associated with prior propensity for exposure to a strong SNI (ATE: − 0.152, *p* = 0.011). The continuous CRP values (data not shown) show that a strong SNI increased the inflammatory levels among women by 60% (ATE: 0.604, *p* = 0.027).

Social network diversity was significantly associated with physical functional limitations. Both the RA and the PSM model showed that women with a high SNI at the baseline had significantly better abilities in both self-reported upper-body (coef.: 0.244, *p* = 0.004; ATE: 0.074, *p* = 0.016) and lower-body functions (coef.: 0.266, p = 0.016; ATE: 0.073, p = 0.004) at follow-up. No statistically significant effect of the SNI on physical functional limitations was detected among men.

Both women and men with a higher SNI at the baseline reported better abilities to perform ADL and IADL tasks. Disabilities in five ADL tasks were associated with a low SNI at the baseline for women in both the RA and the PSM model (coef.: 0.257, *p* < 0.001; ATE: 0.220, p < 0.001) and for men in the PSM model (ATE:0.177, *p* = 0.011). Likewise, the ability to perform IADL tasks was significantly associated with a strong SNI for both women (coef.: 0.660, p < 0.001; ATE: 0.473, p < 0.001) and men (coef.: 0.390, *p* = 0.050; ATE: 0.492, *p* = 0.033).

When regressing the 19 different health outcomes on the instrumented probability of having a strong SNI, the results showed that only cognitive functional impairments were associated with network diversity through the relationship of each respondents’ SNI with his or her level of residential stability. Men with a strong SNI at the baseline had better episodic memory functions at follow-up compared with men with a low SNI (coef.: 4.175, *p* = 0.020). Women with a high SNI, however, scored significantly higher in Raven’s test measuring visuospatial abilities (coef.: 4.983, *p* = 0.028).

## Discussion

To provide additional insights into the causal effects of social networks on older adults’ health, in this study, we appied an outcome-wide [70] multi-method approach [74, 75] to longitudinal panel data to examine prospectively the causal associations of social network diversity with a wider battery of health outcomes among a sub-sample of 3060 Indonesian men and women above the age of 50 years who participated in the fourth and fifth waves of the nationally representative IFLS. Including a large battery of health outcomes and applying three statistical approaches to infer causal inference, this study has produced a large and complex scope of results. To aid the reader, we have organized the discussion section in the following way. First, we shortly review to which extend the three initial hypothesis (gender differences; heterogeneous health effects; effect size gradient along the disablement process) have been confirmed or not. After that, we turn to a more detailed discussion of the significant results (pulmonary pathologies: asthma and lung diseases; immunological impairments: raised CRP values; physical functional limitations and ADL and IADL disability) and situate these within the Indonesian context. Additionally, we try to situate our findings into the overarching framework of the Berkman model and attempt to identify the underlying psychosocial mechanismsm and pathways behind the SNI-health associations in this Indonesian sample of older adults. Since the results from the IV models obviously stand out, we do not only compare the results for functional cognitive outcomes with the existing literature but also discuss the IV method and the role of the instrument in our study. We then turn to strenghts and limitations and conclude with some implications for policy and practice.

### Social network diversity and adult health: gender differences, heterogeneous health effects but no gradients along the disablement process

Using the disablement process model [76] as a paradigm for our analysis of the social network–adult health association, this study found that greater network diversity shows statistically robust associations with various health outcomes along the disablement process. As hypothesized, we identified several gender-specific effects. Women’s social networks exercised a greater health-protecting potential compared to men’s. Social network diversity affected women’s health along the entire disablement process while among men only endpoint disability was affected. In addition, the effect size of the SNI on ADL and IADL disability was overall larger among women compared to men. Many have attempted to explain such social network-related gender differences in health through dispositional or personality differences between men and women [99, 100]. Others, taking a socio-structural approach, have linked the diverging health effects of social networks to the differential impact of socio-economic position, occupational status, and educational background on social network structures and functions among the two genders [101, 102]. Another body of research addressed gender differences by assessing gender-specific social network changes and dynamics across the life course, and particularly assessing aging-related network changes [38]. Particularly, events such as retirement, widowhood or the onset of a chronic disease have differential long-term effects on network size, composition and functional aspects for men and women [1]. In our study, we considered the overall SNI as exposure and did not account for gender-specific differences in the seven SNI components. However, descriptive analyses (data partly not displayed in this study) showed that many of the widowed respondents were women and that re-marriage was common only among men. With advancing age, social activities and thus non-kin ties to neighbors or community members decreased for both men and women but more strongly for women. And with advancing age, more women than men were providing and receiving support from close kin (children, siblings). Furthermore, men and women in our study differed significantly in their educational background (Table 2) which possibly implies a strongly gendered nature of the structural and functional aspects and dynamics of their work and family-related social networks and their effects on health.

As hypothesized, we identified heterogeneous health effects of social network diversity. While greater social network diversity was protective against a range of pulmonary pathologies, functional limitations, and disability outcomes, it also resulted in increased inflammatory levels measured by raised CRP values. This duality has been previously reported by studies focusing also on other inflammatory markers [103] and dysfunctional allostasis [104]. A recent review identified support failures, patterns of rejection or neglect and misdirected control or undermining of healthy practices as three distinct pathways of how negative social exchanges among network members affect health [78]. Again, our aim was to determine whether there are heterogeneous efffects and future studies should look into the exact pathways and mechanisms that link greater social network diversity to increased inflamation or other negative immune responses. As described further below, we in our study hypothesized that for our female respondents network strain from caretaking obligations may play a major role in explaining these heterogeneous health effects.

We did not find support for our last hypothesis with regards to a gradient of social network effects across the disablement process. We post hoc stratified our sample by age groups (50–59, 60–69, 70+ years and pre- vs. post-retirement age of 57 years) but these results did likewise not exhibit a clear gradient in effect size along the disablement process model. To the best of our knowledge, there is no other wide-outcome study using a comparable social network indicator and testing its effect across a range of adult health outcomes to which we could compare our findings. However, some studies testing mediating relationships within the disablement process model have found that main pathway variables (e.g. pathology, impairments, functional limitations) have indirect effects on disability outcomes though different psychosocial factors such as social integration [80], social support [81] or loneliness [79]. These studies conclude that psychosocial factors despite being potential buffers to disablement, their effect along the disablement process is relatively small. These findings may be to some degree comparable to our findings and partly explain the significant effects of social network diversity on ADL and IADL disability but none to limited effects on the preceding main pathway variables. In the future, additional analyses repeating our approach with another social network-related indicators such as network size or distinguishing between different network types could draw a more nuanced picture of potential effect gradients along the disablement process.

### Specific effects along the disablement process and potential mechanisms and pathways

After providing an overall picture of our results, we now turn to a more detailed discussion of the specific health outcomes which were significanlty related to social network diversity. These findings are discussed in light of Berkman et al’s. conceptual framework, which postulates the mechanisms through which social networks affect health [4]. Since the aim of this study was to establish *whether* there are causal effects of network diversity on adult health outcomes and to determine the *size* of these effects, we had only limited possibilities to identify the underlying psychosocial mechanisms and pathways behind these associations. However, in our discussion below, we attempt to provide a few possibilities within the framework of the Berkman model by adding some supplementary results derived from post hoc data stratifications and dissecting the SNI into its single indicators.

### Diverse social networks benefit women’s pulmonary outcomes

The first point to note – limited to women in our study – is the finding of an association between social network diversity and pathologies, that is, pulmonary conditions such as asthma and other chronic lung diseases. This finding is in line with other studies that likewise have provided empirical verification of the relationship between social networks and pulmonary health outcomes. Supportive social networks have been shown to be protective of asthma and other breathing problems, particularly among children [105, 106] and among adult patients with chronic obstructive pulmonary disease (COPD), and social support from social network members has been shown to benefit self-efficacy, treatment adherence, and self-management regimes as well as a number of physical and mental health outcomes [107, 108]. According to Berkman’s model, there are several potential pathways through which a diverse social network may affect respiratory health among Indonesian women. A low SNI among older adult women in our context may be associated with a tendency to remain at home, leading to heightened exposure to indoor triggers of respiratory diseases or lower pulmonary fitness levels, such as allergens, indoor air pollution, or reduced physical activity. Another pathway is through the phenomenon of social influence, which is based on the notion that, when people are connected to others, their behaviours are influenced by them (and vice versa). Social influence flows through networks, and health behaviours like smoking have been found to be “socially transmitted” across social network ties [26]. Data from the World Health Organization from 2016 show that the prevalence of smoking among adults aged 15+ years in Indonesia is high (39%) and that there are large differences between men (76%) and women (3%) [54]. In our data, the prevalence of smoking among women was 8% compared with 77% among men. Our data show further that women with a low SNI were more likely to smoke than women with a high SNI (9% vs 6%, *p* = 0.016; coef.: 0.48, *p* = 0.017); however, for men, the SNI did not affect their smoking status (77% vs 76%, *p* = 0.911; coef.: 0.014, p = 0.911; data not shown). The general religious and socio-cultural norm in Indonesia for women is not to smoke [109, 110]. More diverse networks may be associated with stronger enforcement of this norm; vice versa, the gendered experiences of the smoking stigma may also intensify social isolation, marginalization [111, 112], or feelings of loneliness among Indonesian women [113]. In addition, women are generally perceived as the “caretakers of health” – for their own health and the health of their family [114]. Owing to this, women tend to use more health promotion programmes and have higher health literacy than men. It remains to be tested whether smoking-prohibiting norms and stigmatization are enforced in much stronger ways within women’s social networks, whether there are other qualities present that spread health awareness and resilience to smoking, or whether a combination of factors is at work.

### Diverse social networks negatively affect women’s CRP levels

Our results show significant negative associations between SNI and CRP values, again only among women. Serum CRP levels become elevated in response to acute infections, inflammatory conditions, or trauma and increase with age [115]. A meta-analysis of 83,995 individuals from 14 studies has shown that elevated CRP values can independently predict the risk for all-cause, cardiovascular, and cancer mortality [116], and a literature review of more than 70 studies has shown that social behaviour and inflammation are “intricately connected” [16]. Many studies have indicated that there appears to be a reliable relationship across the life course between social network disruptions in the form of social separation, negative social interactions, loneliness, or widowhood and increased pro-inflammatory activity in terms of an elevated CRP [117], sTNF*α*RII [103], or IL-1Ra and IL-6 [118]. These studies have shown that strong and more diverse social networks increase the odds of receiving instrumental or socio-emotional support, which can either lead directly to lower inflammation (direct-effects hypothesis) or prevent or lighten the effect of stress and thus lower inflammation (buffering hypothesis) [119]. However, our results indicate the opposite, namely that more diverse networks might in fact exercise stress, for example from the burden from or conflict with networks members. Using the SNI as a composite measure of network diversity, we cannot draw a definite conclusion about which exact features of women’s social networks exercise stress, that is, whether stress comes from a larger number of network members or whether some subjective quality plays a role. However, a post hoc examination of the data shows that, after retirement age, there was a stronger negative effect of the SNI on CRP levels among women (ATE: − 0.213, *p* = 0.044). One possible explanation for this may be that retired women – despite having a diverse network – are increasingly facing unmet needs for social support or are notably confronted with growing demands from their network members. Future research should identify the individual or social burdens that lead to strained relationships between network members and affect particularly older women’s health. The literature has suggested that the so-called socially or physiologically defined “elbow points”, namely entering retirement, widowhood, the onset of disease or disability, or informal caregiving obligations, may play a role in the social network–health relationship [1], but this remains to be tested in the Indonesian context.

### Social network diversity positively affects older adults’ physical functioning and disability outcomes

In our study, among women, more diverse social networks were associated with better outcomes in two functional limitation domains measured via six self-reported items of upper- and lower-body functions as well as disability in five ADL and six IADL tasks. Only the ability to perform all ADL and IADL tasks independently was significantly associated with a diverse social network in both men and women, but – except for IADLs in the PSM model – showed stronger associations for women. Our results are generally supportive of our hypotheses and in line with a number of studies that have reported positive associations between network diversity and fewer functional health declines [120] or disabilities in later life [22, 23]. We initially hypothesized to observe stronger effects of social network diversity towards the end of the disablement process, that is, on outcomes that affect respondents’ activities of daily life and their role in society due to increased functional limitations. While we did not idenfity such a gradient in effect size throuhout the whole disablement process, we could still observe a decrease in social network effects along a continuum of severity in disability, ranging from less severe IADL disability related to basic tasks of everyday life to more severe ADL disability related to self-care tasks that allow independent living. However, in the literature, the evidence on the effect of social networks on the different domains of disability among older populations is far from conclusive, as only a few studies have reported on both ADL and IADL outcomes in relation to a social network indicator. We found that our results generally correspond to the results from studies on community-dwelling older adults in Mexico [121] and Spain [122] but contrast with the findings from studies on American [123] and Singaporean older adults [124], which have reported in general more beneficial effects on the performance of ADL tasks. Such mixed findings may reflect the different macro-level framing forces, such as the sociocultural norms and values in which social networks are shaped and function as well as individuals’ experiences and interpretations of these networks and hence the degree to which they can affect their health. In addition, especially when the analyses rely on self-reported information, one may observe cross-cultural differences in the disablement process at large, and particularly the experience of disability might differ across different socio-cultural contexts [125]. Apart from that, the fact that, in our study, social network diversity had a stronger effect on the ability to perform IADL tasks than ADL tasks could be due to several reasons. First, we analysed a relatively young sample (mean age 58 years); thus, more severe ADL disability was less common than IADL disability (12% vs 23%). Further, men suffered less often from ADL limitations than women (10% vs 15%, *p* < 0.001) but reported more disabilities in the performance of IADL tasks (25% vs 22%, *p* = 0.033), which could to some degree explain the null findings in the RA models on ADL limitations for the male sample. The same observation holds for upper- and lower-body functional limitations. Moreover, the ability to perform each of the tasks independently may require very different aspects of one’s social network. For example, ADL tasks related to personal hygiene may require a less diverse network of intimate ties, for example the instrumental help of a spouse, while some IADL tasks can be performed independently through the provision of emotional support from a more distant discretionary network member.

Therefore, while our findings grasp the overall positive impact of social network diversity on both functional limitations and disability among older Indonesian adults, they also raise some important questions about the underlying mechanisms and pathways behind these associations. Particularly, with regard to functional health and disability outcomes, numerous studies have emphasized the need to move beyond crude indicators and further dissect summary measures and discriminate between different types of networks and/or the resources emanating from them, that is, in the form of structural and functional support [35, 126]. It has also been noted that the failure to do so has possibly led to a number of null findings showing that social networks were not related to disability outcomes [127]. Previous studies have shown that family-based networks, that is, relationships with the spouse and adult children, are the most frequent network types in which older adults are embedded [128]. Family members are the first ones to turn to in need of immediate assistance and should therefore play an important role when it comes to influencing functional limitations or disability outcomes in old age. Studies have shown that family-based networks are protective against the onset of disability and promote recovery [129, 130]. However, the evidence on spousal and parent–child (ren) relationships offers very heterogeneous results. Some studies have found that childlessness can have positive effects on old-age mobility but that relationships with co-residing children can increase the risk of future disability [131]. Additionally, more nuanced analyses of spousal relationships have differential effects on mobility impairment and disability depending on whether the spouse provides emotional support, which facilitates improvement, or instrumental support, which obviates the overcoming of limitations [131]. Again, using a crude indicator, we cannot draw any definite conclusions about which network types or which structural or functional aspects of older adults’ social network play a more important role in functional limitations and disability outcomes. Overall, though, the SNI in this study had a strong family-based focus, and five out of seven indicators were related to family members. Our results show that particularly women receive a better health impact from a more diverse social network than men, and there are indications in the data that the marital dyad and ties to adult children seem to play an important role in older women’s health. In fact, when replacing the crude SNI post hoc with single social network indicators (data not shown), for women, the presence of a spouse had a protective effect on functional limitations and disability, while support from or to children in our study was negatively associated with reduced lower-body functions. Recent censuses and surveys have shown that many widowed older adults are women and that remarriage seems to be a more common practice among widowed men than among women [41]. Due to the country’s demographic and economic transitions, women in Indonesia are facing a host of challenges that inter alia contribute to “holes” in older women’s social networks. For example, having a longer life expectancy, many women outlive their husband and many continue to live in low-income single-person households in predominantly rural areas; in addition, due to fertility declines and increasing rural-to-urban labour migration, fewer adult children are available to elderly relatives as caregivers. In Indonesia, but also in other LMICs undergoing similar developments, such transitions are putting immense pressure on the traditional informal support systems of older adults, and policies should be responsive to these trends and find ways to “fill” these network holes, as they have strong implications for health, particularly among vulnerable segments of society, such as women, especially women who intersect with other macro-contexts, such as poverty or rurality, because they have the fewest opportunities to counterbalance the effects of small and less diverse networks on their health.

### Greater network diversity benefits older adults’ cognitive health

Our last finding to be discussed relates to the results from the IV models showing a strong association of the SNI with cognitive performance. After instrumenting for residential instability, a factor that we – based on the review of the literature (e.g. [96, 132]) – appraised as a potential barrier to social network development, strong associations between social network diversity and cognitive outcomes remained; that is, men and women with a higher SNI reported better episodic memory and visuospatial abilities, respectively. From a theoretical perspective, there are clear reasons to expect an association between social networks and cognitive performance outcomes (for reviews, see [18, 34]). The cognitive reserve hypothesis, the vascular hypothesis, and the stress hypothesis are three major aetiological hypotheses that have been proposed to be the most relevant to the preservation of cognitive abilities [34]. A systematic review evaluating the association between different aspects of social relationships with the cognitive functioning of healthy older adults has summarized evidence from 39 studies and suggested relationships between social activity participation and processing speed and visuospatial abilities and between social support and composite measures of social relationships and episodic memory [18]. In our study, we had limited capacity to identify the psychosocial mechanisms or pathways that eventually associated the instrumental probability of a high SNI with better outcomes in episodic memory and visuospatial abilities among men and women, respectively. One could suspect – following Berkman’s framework – that more diverse networks provide more opportunities for social interactions and higher social engagement enhances cognitive reserves. Such a build-up of cognitive reserves allows for more efficient use of neural networks and thus enables better visuospatial abilities. The stress hypothesis, however, may be more useful for explaining the results relating to episodic memory. Many studies have outlined the stress-reducing benefits of social support, and lower levels of stress have been shown to benefit memory performance (for reviews, see [18, 34]).

Of theoretical interest is also that the degree of association between social network diversity and cognitive performance decreased along the continuum from fluid (i.e. visuospatial ability, coef.: 4.983) to crystallized intelligence (episodic memory, coef.: 4.175). The opposite has been observed in a Swedish study [133]. To the best of our knowledge, this is the first study to report results on fluid and crystallized intelligence in relation to social networks for an older LMIC population. Further, we again observed distinct gender differences that could be interpreted *vis à vis* the role of the instrument in the Indonesian context. Censuses have shown that, in Indonesia, many older adults migrate, that is, change their residency due to mortality or work [41]. In many cases, women, after the death of their husband, move to be near their adult children and may face a range of challenges (and cognitive demands) stemming from social network changes, namely the disruption of the old network and building up of new ties, adapting to a new physical environment, and possibly new duties, for example taking care of grandchildren. Labour migration and job mobility, on the other hand, seem to be the prime reason for men to change their place of living in many cases. In fact, in our study (data partly shown in Table 2), men moved more often than women (11% vs 7%, *p* < 0.001) and increasingly before reaching retirement age (15% vs 7%, *p* < 0.001). Further, among those who changed their residency in our study, more women than men reported being single and thus were most likely to be widowed (27% vs 9%, *p* < 0.001).

Another issue to be discussed in regard to the IV models is the large number of null findings for the other health outcomes. We believe that this can likewise be explained by the role of residential stability in the Indonesian setting rather than by potential model misspecifications. A common assumption in IV analyses is deterministic monotonicity, meaning that, while the instrument may have no effect on some people, all those who are affected are affected in the same way [134]. Such an assumption, however, sometimes does not prove to be realistic. We assumed that any move within the past 7 years would represent a disruption in people’s social network. However, the opposite may be true in some cases. In this study, we did not consider motivations for moving, how far or to where IFLS respondents moved between the years 2000 and 2007/08. In addition, the IFLS data do not contain the reasons for moving or information about to whom people moved. It could have been the case that participants moved only a short distance, which enabled them to stay in touch with their old network while at the same time building up a new one. Even a long-distance move might not have a negative effect if the participants returned to a familiar setting or reconnected with family, friends, or other acquaintances who already resided in the new location. Possibly, the new location could also offer a better network support than the old one. During the first half of 2000, social media expanded in Indonesia, and sustaining access to previous social networks through social media can attenuate the effects of migration. Recent surveys have shown that, in 2018, 50% of all adults over 50 years of age owned a mobile phone; smartphone ownership increased from 3% in 2013 to 13% in 2018 [135]. Furthermore, labour migration is increasingly common in Indonesia following the Asian economic crisis. Moving to a new place for economic reasons, such as a prospective job, may have benefits by itself that outweigh the potential harmful effect of disconnecting from old network members. Further, in a very collectivist society like Indonesia, one move within 7 years may not have the same effects as in a less-collectivist Western society.

### Methodological considerations, strengths and limitations

In this study, we used a comprehensive and large panel data which allowed us to employ multiple analytical strategies to address potential treatment biases. However, performing RA, PSM and IV techniques led to discrepant results – a situation which overall demonstrates the difficulty of determining causal inference in observational studies. Our results show that the estimated associations between social network diversity and various adult health outcomes are sensitive to the choice of analytical method. There was general concordance between the RA and PSM models, which is also commonly observed in other studies [136]. However, between the RA/PSM and IV models there was no concordance and the benefits of social network diversity on physical health disappeared in the IV adjusted models but yielded effects for two cognitive health outcomes. With a rising number of studies simultaneously applying multiple methods, such discrepancies between PS and IV analyses are not infrequent in medical and public health research [137]. Still, the question remains why the IV models did not yield the same picture as the RA and PSM models. When results are conflicting, many tend to take the IV results as the “true” estimations because of the ability of IV analyses to account for unmeasured or unkown confounders in addition to the measured ones. Here, we do not unquestioningly consider the IV estimates as our true results but instead argue that both PSM (and RA) and IV estimates despite yielding differences are also simultaneously correct. As outlined further above, both PSM and IV techniques were systematically implemented and relied on reliable propensity scores with a good support scenario (Appendix 1) and an IV which has been theoretically and statistically validated (tests of endogeneity: Durbin (*p* = 0.049), Wu–Hauser (*p* = 0.045); instrument strengths F = 19.58 (*p* = 0.002); see Fig. 2c). Considering the conceptual differences between the two methods may aid in interpreting the discrepant results. While the PSM technique produces average treatment effects (ATE), the IV results should be interpreted in terms of local average treatment effects (LATE) restricted to a group of marginal respondents. These marginal respondents are a subset of respondents whose exposure choices (i.e. varying social network diversity) are affected by variations in the IV measured by residential (in) stability [73]. Under the assumption that treatment/exposure effects are heterogeneous and the exposure assignment is related to this heterogeneity (i.e. there is evidence suggesting that certain subgroups of respondents are more prone to benefit from greater social network diversity), the ATE and the LATE are different estimations. Thus, despite yielding discrepant results, both estimates are simultaneously correct [137]. As mentioned earlier, by applying both confounder control and instrument-based approaches, we are able to draw conclusions both on individual and population level since these techniques produce effect estimates which apply to different populations [72, 73]. While the RA and PSM models provide insights into the specific effects of social network diversity on various health outcomes for individual respondents, the IV models measure effects for a “marginal” population which excludes those respondents who would “always” or “never” have a diverse network independently of their residential stability and focuses on respondents whose likelihood of having a high SNI depends on their state of residential stability. Since many decisions with regards to changing residence in old age are less bound to individual-level characteristics but more often context-related, the IV results can guide social and health policies that relate to late-life moving, relocations, geographic mobility and migration among aging populations and more broadly to issues relating to “aging in place”, and the effects of social network changes and network turnover on older adults’ cognitive health.

This study advances the prior literature in several ways. First, we took an outcome-wide analytic approach to provide a comprehensive picture of the role of social network diversity across the disablement process. This is the first study to present such evidence for an Indonesian sample, and it helps in synthesizing the previously scattered evidence on single outcomes from other studies. Second, the application of multiple causal inference methods on a longitudinal panel data, which established the temporal order of exposure and outcome, and extensive covariate and baseline health control to reduce the option of reverse causation altogether permitted more evidence for a robust causal interpretation of our results.

The present study is, however, still subject to certain limitations, some of which may be dealt with in future research. Many outcomes (13 out of 19) and the exposure in this study are based on self-reported information and thus might be subject to social desirability or common method bias. While most outcomes were measured for all the respondents, the biomarkers CRP and HbA1c were only measured for a sub-sample, which could reduce the precision of those models. Further, biomarkers are based on DBS testing and may therefore not be comparable to studies using assay results from venous blood.

While we took guidance from established approaches in the calculation of the SNI, we also performed some modifications to it in our study which may make it difficult to compare our findings directly with other studies employing a measure of social network diversity. One limitation of our SNI may derive from the inclusion of household size as one of the seven SNI indicators. This leads to potential double counts of existing networks in the SNI and makes the different types of networks non-exclusive. While this approach entails some limitations, we still deemed it valuable to include the household size measure into our SNI in order to account for the multi-ethnic and customary diverse setting of Indonesia where various living and cohabitation combinations exist. The male-headed nuclear household is not the default setting across the Indonesian setting and we thus believe there is added value in incorporating houshold size into our SNI to account for networks that go beyond the spousal dyad or other co-residing close kin.

Further, self-selection bias may be a concern in our study. As mentioned above, we do not account for any details of mobility patters between IFLS-3 and IFLS-4 nor do we account for the reasons for changing residence or perform comparisons between characteristics of the old and new area of residence. It might be possible that health status or health-related attitudes and behaviors predict the decision to change residence and the choice of destination in our study. Estimating the magnitude of self-selection bias in observational studies remains a methodological challenge and it has been noted that self-selecion may potentially inflate the observed associations [138]. Future studies should more closely examine the role of self-selection, particularly residential self-selection in health-related social network studies.

Lastly, our findings also need to be interpreted in the light of the vast ethnic diversity in Indonesia (300+ ethnic groups), which may have different patterns of social networks and a varied structural significance of gender. On the other hand, the fact that we tested the relationships between network diversity and health in a large socio-culturally diverse sample strengthens the generalizability of our findings across populations. Despite these limitations, our findings offer a better understanding of the role of social networks and network diversity in the disablement and aging process in Indonesia and offer an opportunity for future studies to investigate additional aspects of social networks in relation to adult health and aging processes.

## Conclusions

This study considered the social networks of older Indonesian adults, a population for which, to the best of our knowledge, only a few prior studies have been conducted. We examined the effect of social network diversity on a large battery of 19 health outcomes representing disablement and aging processes, and our findings suggest that the ability to call on a diverse set of social networks confers strong heterogeneous long-term health effects, particularly for older women. Due to its outcome-wide approach, this study can convey useful information to different groups of policy audiences. For instance, the findings on social networks’ role in shaping chronic disease outcomes, including the potential role for smoking interventions as well as results pertaining to various impairments, will be useful for policy makers who are involved with primary and secondary prevention efforts. There should be room in future health polices to provide a framework to integrate patients’ social network members into disease treatment and particularly NCD management schemes, particularly among older populations and for diseases that require more thorough monitoring and self-management. On the other hand, the results concerning functional limitations and disability will be useful for decision makers who hold responsibilities for tertiary prevention, such as shaping social and health policies related to the promotion of aging in place, the provision of chronic long-term care, disability rehabilitation, and future health care and geriatric care management. The crucial role of social networks in shaping adult health outcomes should arguably be considered in various health promotion programmes and translated into multilevel interventions and intersectoral health and welfare policies. Overall, our study highlights the need for gender-specific policies, because women gain greater health protection from their social networks than men. Interventions should particularly be designed to strengthen, that is, mobilize or optimize, older adults’ social networks at times of certain “elbow points”, specifically retirement, widowhood, or the onset of disease or disability. Besides gender, particularly in an LMIC setting, policies should have sensitive intersections with poverty and rurality, as these are contexts in which formal structures, that is, health care services, are relatively weak and in which informal structures, that is, personal social networks, are relatively strong.

## Figures and Tables

**Fig. 4:**
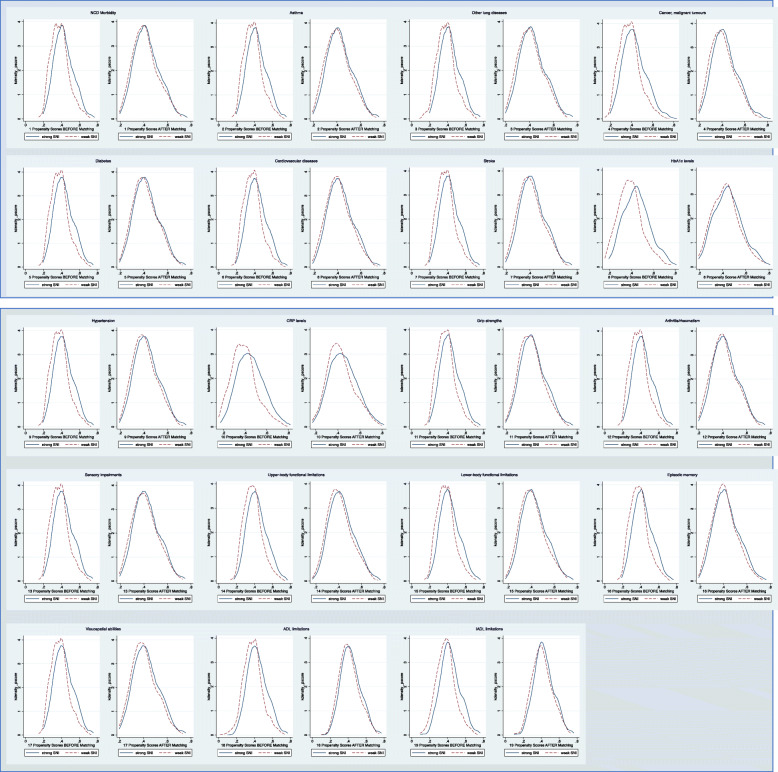
Balance plots showing the distribution of the propensity scores for the exposed (high SNI) and control groups (low SNI) before and after matching. Source: IFLS

**Fig. 5:**
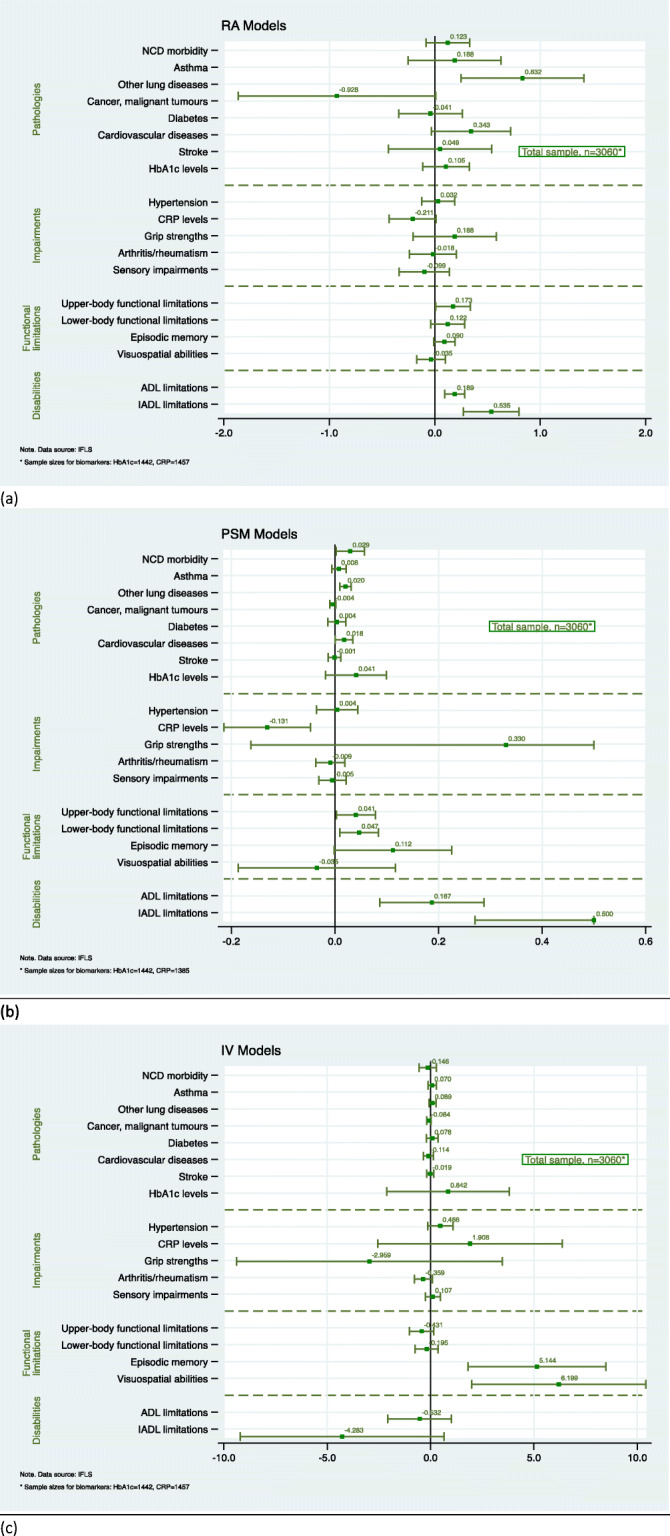
Coefficient plots displaying the results from (**a**) regression adjustment (RA), **b** propensity score matching (PSM), and (**c**) instrumental variable analysis models (IV) for effects of baseline SNI on various subsequent adult health outcomes for the total sample. Source: IFLS

## Data Availability

The IFLS-4 and IFLS-5 datasets that were analysed during the current study and which support the findings of this study are publicly available upon registration from the RAND Corporation: https://www.rand.org/well-being/social-and-behavioral-policy/data/FLS/IFLS.html

## Appendix 3

**Table 4:** Results from regression adjustment (RA), propensity score matching (PSM), and instrumental variable analysis models (IV) for effects of baseline SNI on various subsequent adult health outcomes for the total sample and stratified by sex/gender

Health outcomes	Total sample (***n*** = 3060)						Women (***n*** = 1583, 52%)						Men (***n*** = 1477, 48%)					
	RA		PSM		IV		RA		PSM		IV		RA		PSM		IV	
	*Coef*	*p*	*ATT*	*p*	*LATE*	*p*	*Coef*	*p*	*ATT*	*p*	*LATE*	*p*	*Coef*	*p*	*ATT*	*p*	*LATE*	*p*
NCD morbidity	0.12	0.411	0.029	0.480	−0.146	0.499	0.26	0.126	0.029	0.052	−0.672	0.447	−0.04	0.633	0.004	0.904	−0.013	0.950
Asthma	0.19	0.410	0.008	0.383	0.070	0.502	**0.69**	**0.018**	**0.023**	**0.002**	−0.118	0.729	− 0.34	0.196	− 0.007	0.171	0.146	0.196
Other lung diseases	**0.83**	**0.002**	**0.020**	**0.001**	0.089	0.315	**1.07**	**0.009**	**0.020**	**0.000**	− 0.019	0.940	0.63	0.103	0.013	0.064	0.111	0.262
Cancer, malignant tumours	**−0.93**	**0.050**	− 0.004	0.082	− 0.084	0.123	− 0.95	0.142	− 0.006	0.179	− 0.165	0.424	−0.87	0.213	−0.006	0.234	−0.062	0.224
Diabetes	−0.04	0.296	0.004	0.370	0.078	0.590	0.04	0.517	0.006	0.882	0.389	0.478	−0.13	0.357	0.010	0.985	−0.039	0.792
Cardiovascular diseases	**0.34**	**0.048**	0.018	0.134	−0.114	0.362	0.27	0.177	0.014	0.104	−0.165	0.669	0.43	0.128	0.016	0.224	−0.103	0.427
Stroke	0.05	0.844	−0.001	0.862	−0.019	0.829	−0.09	0.859	−0.001	0.805	−0.538	0.309	0.12	0.774	−0.009	0.605	0.129	0.223
HbA1c levels	−0.10	0.988	0.041	0.837	0.842	0.829	0.18	0.970	0.060	0.732	−1.202	0.492	0.04	0.972	−0.030	0.770	0.052	0.927
Hypertension	0.03	0.155	0.004	0.115	0.466	0.138	−0.08	0.594	−0.025	0.914	0.981	0.408	0.12	0.232	0.026	0.201	0.301	0.331
CRP levels	−0.21	0.063	**−0.131**	**0.002**	1.908	0.746	−0.09	0.247	**−0.152**	**0.011**	−1.249	0.480	−0.32	0.192	−0.065	0.214	0.088	0.351
Grip strengths	0.19	0.364	0.330	0.068	−2.959	0.370	0.07	0.806	−0.260	0.890	−6.162	0.524	0.38	0.246	0.464	0.326	−2.099	0.578
Arthritis/rheumatism	−0.02	0.808	−0.009	0.695	−0.359	0.113	−0.05	0.717	0.003	0.739	−1.061	0.350	0.07	0.910	0.008	0.627	−0.177	0.343
Sensory impairments	−0.09	0.576	−0.005	0.660	0.107	0.563	−0.12	0.531	−0.023	0.271	0.564	0.455	−0.04	0.990	−0.010	0.740	0.014	0.938
Upper-body functional limitations	**0.17**	**0.014**	**0.041**	**0.011**	−0.431	0.157	**0.24**	**0.004**	**0.074**	**0.016**	−1.261	0.380	0.18	0.199	0.032	0.364	−0.208	0.427
Lower-body functional limitations	0.12	0.166	0.047	0.133	−0.195	0.492	**0.27**	**0.016**	**0.073**	**0.004**	−0.450	0.643	−0.07	0.487	0.004	0.854	−0.163	0.551
Episodic memory	0.09	0.062	0.112	0.206	**5.144**	**0.002**	0.06	0.283	0.043	0.523	6.404	0.051	0.12	0.130	0.070	0.207	**4.175**	**0.020**
Visuospatial abilities	−0.04	0.967	−0.035	0.855	**6.199**	**0.003**	−0.12	0.266	−0.080	0.465	**4.983**	**0.028**	0.08	0.208	0.063	0.193	7.728	0.058
ADL limitations	**0.19**	**0.000**	**0.187**	**0.000**	−0.532	0.501	**0.26**	**0.000**	**0.220**	**0.000**	−5.053	0.334	**0.10**	**0.112**	**0.177**	**0.011**	0.842	0.270
IADL limitations	**0.56**	**0.000**	**0.500**	**0.003**	−4.283	0.096	**0.66**	**0.000**	**0.473**	**0.000**	−13.602	0.327	**0.39**	**0.050**	**0.492**	**0.033**	−1.812	0.432

Note: *Coef* Regression coefficients, *ATT* Average treatment effects, *LATE* Local average treatment effects, *p p*-value (significant results are printed in **bold**)

## Abbreviations

ADLs: Activities of daily living
ATE: Average treatment effect
BMI: Body mass index
BP: Blood pressure
Coef: Coefficient
COPD: Chronic obstructive pulmonary disease
CRP: C-reactive protein
CVDs: Cardiovascular diseases
DBS: Dried blood spots
ELISA: Enzyme-linked immunosorbent assay
HbA1c: Glycated haemoglobin
HICs: High-income countries
IADLs: Instrumental activities of daily living
IFLS: Indonesian Family Life Survey
IL-1Ra and IL-6: Interleukin-1 receptor antagonist and interleukin-6
IV: Instrumental variable
LBFL: Lower body functional limitations
LMICs: Low and middle-income countries
NCDs: Non-communicable diseases
PCE: Per capita expenditure
PSM: Propensity score matching
RA: Regression adjustment
SD: Standard deviation
SNI: Social network index
SRH: Self-rated health
sTNF*α*RII: Soluble tumour necrosis factor- *α* receptor II
UBFL: Upper body functional limitations
UHC: Universal Health Coverage
VIF: Variance inflation factor
WHO: World Health Organization
